# PrEP Awareness and Attitudes in a National Survey of Primary Care Clinicians in the United States, 2009–2015

**DOI:** 10.1371/journal.pone.0156592

**Published:** 2016-06-03

**Authors:** Dawn K. Smith, Maria C. B. Mendoza, Jo Ellen Stryker, Charles E. Rose

**Affiliations:** Division of HIV/AIDS Prevention (DHAP), National Center for HIV, Viral Hepatitis, STD, and TB Prevention (NCHHSTP), Centers for Disease Control and Prevention (CDC), Atlanta, Georgia, United States of America; Asociacion Civil Impacta Salud y Educacion, PERU

## Abstract

**Objectives:**

As trials were assessing the safety and efficacy of daily oral antiretroviral preexposure prophylaxis (PrEP) for the prevention of HIV infection, there was a clear need to understand the evolution of knowledge of, and attitudes toward, PrEP among primary care clinicians.

**Methods:**

Physicians and nurse practitioners were surveyed in 2009 (n = 1500), 2010 (n = 1504), 2012 (n = 1503), 2013 (n = 1507), 2014 (n = 1508) and 2015 (n = 1501) to assess their awareness of PrEP, willingness to prescribe PrEP, and whether they support use of public funds to pay for PrEP. Pharmacists (n = 251) were surveyed about PrEP in 2012 only. Descriptive statistics were computed for physician demographics and PrEP-related questions. Prevalence ratios for willingness to prescribe PrEP were computed using Poisson regression analysis.

**Results:**

Awareness of PrEP was low among clinicians (2009: 24%, 2010: 29%) but increased after trials reported effectiveness (2012: 49%, 2013: 51%, 2014: 61%, 2015: 66%). Following a description of PrEP with an estimated effectiveness of 75%, across 6 of the study years 91% of clinicians indicated a willingness to prescribe PrEP to at least one group at high risk of HIV acquisition. A smaller majority of clinicians indicated support for public funding of PrEP in 2009: 59%, 2010: 53%, and 2013: 63%.

**Conclusions:**

In surveys conducted before and after the release of PrEP trial results, primary care clinicians were largely unaware of PrEP. They indicated high levels of willingness to prescribe it for patients at high risk of HIV acquisition and expressed interest in education about how to deliver this new clinical HIV prevention method. It will be important to continue monitoring clinician knowledge, attitudes, and practices as the use of PrEP increases in the US.

## Introduction

In recent years, reductions in rates of new HIV infections in the United States (US) have stalled at approximately 45,000 per year and in some groups (e.g., young men) annual HIV infections are increasing [1]. In response to the need for additional effective prevention methods, clinical trials were undertaken to test the safety and effectiveness of daily oral antiretroviral preexposure prophylaxis (PrEP). Between 2010 and 2014, these trials reported the effectiveness of daily doses of tenofovir disoproxil fumarate (TDF) alone or in combination with emtricitabine (FTC), in reducing HIV acquisition among gay, bisexual, and other men who have sex with men (MSM) [2], persons who inject drugs (PWID) [3], and heterosexually-active men and women (HET) [4,5].

In parallel to the start of efficacy trials, efforts began to understand how PrEP would best be implemented in the US when and if safety and substantial efficacy were demonstrated. A few studies were done to assess early levels of knowledge about, acceptability of, and use of PrEP in selected US groups of MSM [6–9] and heterosexuals in care at sexually transmitted disease (STD) clinics [10, 11]. However, few studies were done early on among the primary implementers of this new intervention, clinicians who would be called upon to identify their HIV-uninfected patients for whom PrEP would be most appropriate and to prescribe and manage a course of PrEP medication with indicated counseling and safety monitoring [12–16]. These early clinician studies were limited to a single geographic area, or to HIV or infectious disease specialists.

We set out to assess the awareness of, and attitudes about, PrEP in a national sample of primary care clinicians in the US. Given the vigorous recent public conversations about health care costs and the roles of public and private sector payers, we also assessed some attitudes about the acceptability of public funding for PrEP.

## Materials and Methods

### Survey Methods

Porter Novelli Public Services conducts annual web-based surveys with a main sample of primary care physicians and additional samples of other selected specialties. In 2009, 2010, and 2012, respondents for the DocStyles surveys were drawn from the Epocrates opt-in, verified panels of physicians (Honors Panel) and nurse-practitioners and registered dieticians (Allied Health Panel). Verification was achieved by checking each physician's first name, last name, date of birth, medical school, and graduation date against the American Medical Association’s master file of physicians licensed in the United States at the time of panel registration. A random sample of clinicians—drawn each year to match the American Medical Association's master file proportions for age, sex, and region—were invited to participate in the survey. Physicians, nurse practitioners, and registered dietitians were then screened for eligibility to participate in the survey if they practiced in the United States; actively saw patients; worked in an individual, group, or hospital practice; and had practiced medicine for at least 3 years. Pharmacists were included in 2012 only and were required to be customer-facing, work in the United States, and have been a pharmacist for at least three years. Quotas were set each year to reach 1,000 primary care physicians, 250 pediatricians, 250 OB/GYNs, 250 nurse practitioners, 200 registered dietitians, and in 2012 only, 250 retail pharmacists.

In 2014 and 2015, the clinician samples were drawn from SERMO’s Global Medical Panel which includes over 330,000 healthcare professionals. Panelists were verified using a double opt-in sign up process with telephone confirmation at place of work. Each year, SERMO took a random sample of eligible healthcare professionals in the United States from their main database to load into their invitation database.

In all survey years, pediatricians and registered dieticians were not asked about PrEP on the DocStyles questionnaire and therefore are not included in our analysis sample. In 2009, the only year they were included in DocStyles, dermatologists were excluded from PrEP questions and the analysis sample.

Survey instruments were developed by Porter Novelli with technical guidance from its federal public health agencies, nonprofit, and for-profit clients. Some survey questions were not asked in all survey years. In the first two survey years (2009, 2010), attitudes were assessed based on a hypothetical PrEP effectiveness of 75% because these survey waves were conducted prior to the publication of results from any of the PrEP effectiveness trials. In the three trials that showed significant reductions in the risk of sexual acquisition of HIV infection among those given PrEP [2, 4, 5], the prevention effectiveness shown for trial participants with high self-reported adherence (73–78%) was very similar to the estimate provided to the DocStyles survey respondents (75%). This estimate was especially appropriate given that, in clinical practice, medication adherence is most commonly assessed by patient self-report. Based on additional data about the effectiveness of PrEP among those with drug detected indicating adherence to daily doses, the data presented in the survey was more nuanced. In 2012 and 2013, the estimated effectiveness provided to respondents was “90% or more among those who took it nearly every day” and less if adherence was poor (2012; 0–50%, 2013; 20–50%).

The Centers for Disease Control and Prevention (CDC) licenses access to results of the DocStyles surveys post-collection from Porter Novelli. Analysis of these data was exempt from CDC institutional review board approval because personal identifiers were not included in the data files received for analysis.

### Analysis Methods

Prevalence ratios were computed to determine the strength of association between clinicians willing to prescribe PrEP to at least one high risk group (PWID, MSM, persons with an STD, people who change sex partners frequently, HIV discordant couples, HIV discordant couples attempting to conceive) and each of eight clinician characteristics (age, sex, race/ethnicity, clinician specialty, clinician work setting, number of clinicians in practice, number of years in practice, having provided antiretrovirals for postexposure prophylaxis (PEP) or treatment of HIV infection).

Univariate prevalence ratios measured the association between the outcome and each of the eight characteristics. Multivariable prevalence ratios measured the association between the outcome and each of the eight characteristics adjusting for the effects of all other characteristics in a multiple regression model.

Generalized estimating equations using a robust variance estimator and assuming a Poisson model with an independent working correlation matrix was used to analyze the effect of time (measured in years) on the proportions of clinicians who have heard of PrEP, who are willing to prescribe PrEP to at least one high risk group, and to each high risk group. High risk groups identified were PWID, MSM, persons with a sexually transmitted disease, people who change sex partners frequently, HIV discordant couples not intending conception, and HIV discordant couples attempting to conceive. All eight characteristics and each of their interactions with year were considered in adjusted linear trend analyses. Both forward selection and backward elimination were used to determine the factors that were controlled for in each model.

Only those characteristics and those interactions that were significant at the 0.05 level were kept in the final models.

## Results and Discussion

For the 2009 survey, of the 2,825 eligible physicians (excluding dermatologists and pediatricians) invited, 1,250 (44%) physicians completed the entire survey. Of 500 eligible nurse-practitioners invited, 250 completed the entire survey (response rate 50%). For the 2010 survey, of the 2,308 eligible physicians invited (excluding pediatricians), 1,250 (54%) completed the entire survey. Of 431 eligible nurse-practitioners invited, 254 (59%) completed the entire survey. For the 2012 survey, of the 2,664 eligible physicians invited (excluding pediatricians), 1,251 (34%) completed the entire survey. Of 456 eligible nurse-practitioners invited, 252 (55%) completed the survey. For the 2013 survey, of the 1,802 eligible clinicians invited, 1,257 (70%) completed the survey. Of the 450 eligible nurse practitioners invited, 250 (55%) completed the survey. For the 2014 survey, of the 1717 eligible clinicians invited, 1258 (73%) completed the survey. Of the 398 eligible nurse practitioners invited, 250 (63%) completed the survey. For the 2015 survey, of the 1,794 eligible clinicians invited, 1,500 (84%) completed the survey. Of the 481 eligible nurse practitioners invited, 251 (40%) completed the survey.

### Demographic and Practice Characteristics of Responding Clinicians

The majority of clinicians were male (61%), white (70%), and worked in a group practice (66%) (Tables 1 and 2). Family practitioners/general practitioners (36%) and internists (31%) constituted the largest groups of respondents, followed by nurse practitioners (17%) and obstetrician/gynecologists (17%). Among clinicians, 66% believed the population served by their practice was low prevalence for HIV (<1%); 16% believed it to be a moderate prevalence population (1–5%) and 3% a high prevalence population (>5%). Furthermore, 15% responded they did not know the level of HIV infection in their practice community.

**Table 1:** Clinician Characteristics, DocStyles, 2009–2015, United States. Total Sample Size (2009–2015) N = 9023.

Characteristic	Median value	Range
Age (years)	46	22–95
Number of Clinicians in Practice	5	1–999
Number of Years in Practice	14	3–50

**Table 2:** Clinician Sample Characteristics, DocStyles, 2009–2015, United States. Total Sample Size (2009–2015) N = 9023.

Clinician Sample Characteristics	%
Sex	
Male	60.9
Female	39.1
Race/Ethnicity	
White	69.7
Black/African American	3.2
Hispanic/Latino	4.2
Asian	17.6
Other	5.4
Specialty	
Family Practice/General Practitioner	36.1
Internist	30.6
Obstetrician/Gynecologist	16.6
Nurse Practitioner	16.7
Work Setting	
Hospital Inpatient Practice	13.9
Group Outpatient Practice	66.4
Individual Outpatient Practice	19.7
Region (2012–2015 only)	
Midwest	22.9
Northeast	25.4
South	30.2
West	21.4

### HIV Testing Practices

Routine screening for HIV infection was reported by 35% of clinicians for all sexually active adults not previously tested and by 20% for all patients (ages 13–64) not previously tested. Most (81%) clinicians reported providing HIV testing when requested by a patient. Condition or population-specific routine HIV testing was reported by 74% of clinicians for patients seeking treatment for an STD, by 55% for all pregnant women, by 52% for all MSM, and by 36% for all patients initiating treatment for tuberculosis. A minority (9%) do not offer routine HIV screening for any group of patients.

### Antiretroviral Prescribing

In 2012 and 2013, an average of 26% of clinicians responded that they had prescribed antiretroviral medication for treatment of HIV infection; among these clinicians, 44% reported providing treatment for 1–4 HIV-infected patients, 24% for 5–9 patients, 18% for 10–24 patients, and 15% for 25 or more patients.

Across six survey years (2009, 2010, 2012, 2013, 2014, 2015), 24% reported having prescribed antiretroviral medication for occupational postexposure prophylaxis (PEP) and 10% had prescribed it for nonoccupational PEP. In 2009, 2010, and 2012, few clinicians (1%) reported having prescribed PrEP. The proportion reporting having prescribed PrEP rose to 4% in 2013, remained 4% in 2014, and rose to 7% in 2015. In 2015, of those who had prescribed PrEP, 73% had done so for MSM, 22% for PWID, 22% for uninfected men and 27% for uninfected women in HIV discordant couples during conception attempts, 45% for uninfected women in an HIV discordant couple not planning conception and 30% for uninfected men in this situation.

### Awareness and Attitudes About PrEP

Awareness of PrEP was low in 2009 (24%) and 2010 (29%) but increased to 49% in 2012, 51% in 2013, 61% in 2014, and 66% in 2015 (adjusted p-value of <0.001). In 2013, few reported having read CDC interim guidance for PrEP use with MSM (8%), heterosexually active adults (10%) or PWID (10%). However, in 2014, 17% reported having read the CDC’s PrEP clinical practice guidelines published in May of that year.

Across five survey years (2009, 2010, 2012, 2013, 2015), the majority of clinicians supported PrEP use for one or more risk populations presented (91%). Clinicians’ willingness to provide PrEP was highest for the uninfected partner in an HIV discordant couple (79%), followed by MSM (66%) and PWID (63%), those in HIV discordant couples planning conception (61%), persons who change sexual partners frequently (56%), and persons with a diagnosed STD (34%).

**Fig 1:**
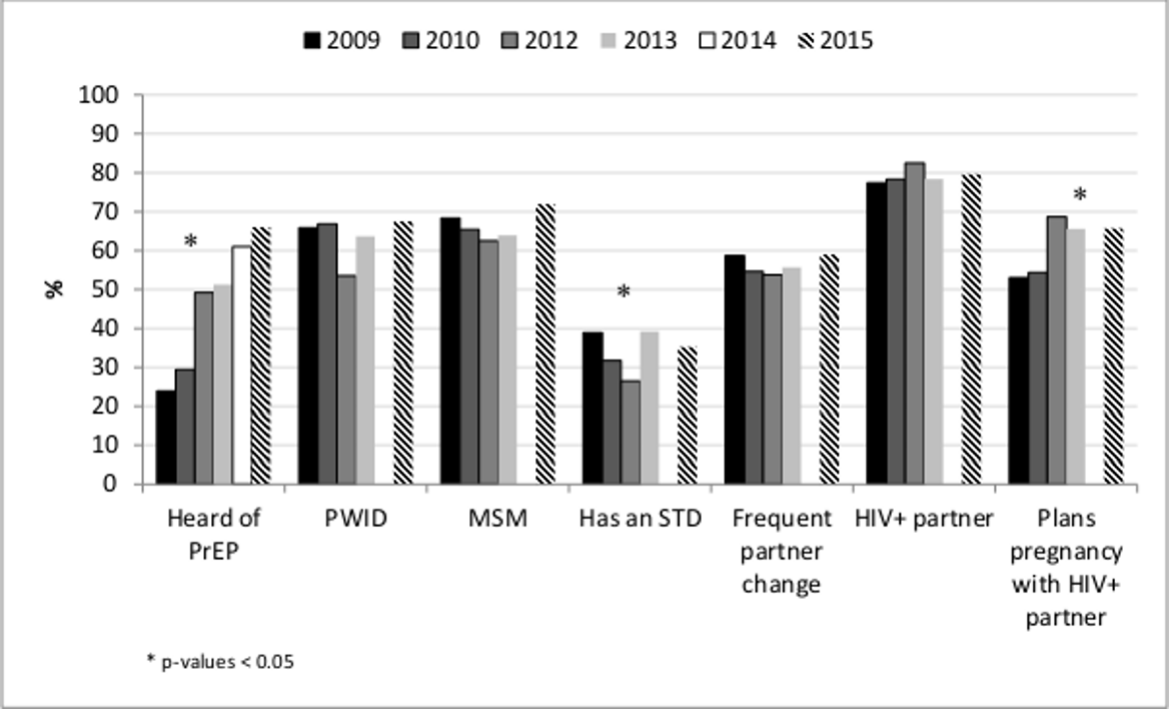
Awareness of PrEP and Willingness to Prescribe PrEP to Persons with Selected Risks for HIV Acquisition by Survey Year, DocStyles, 2009–2015, United States.

**Fig 2:**
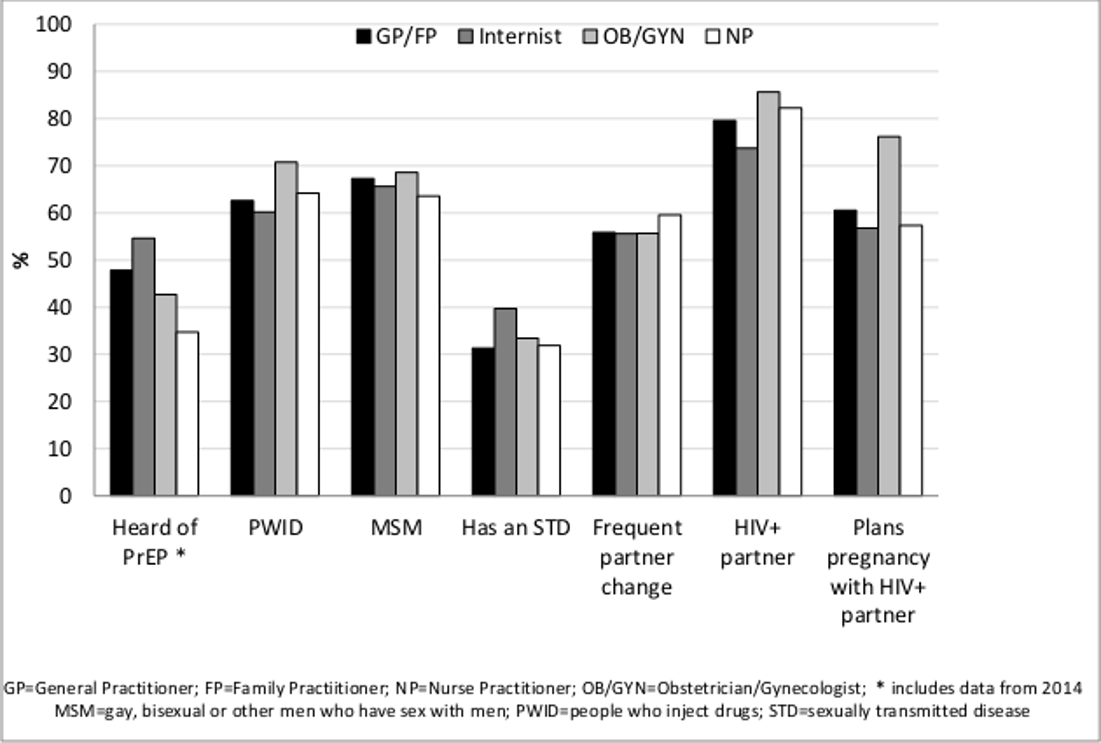
Awareness of PrEP and Willingness to Prescribe PrEP to Persons with Selected Risks for HIV Acquisition by Provider Type, DocStyles, 2009–2015, United States.

Willingness to prescribe for at least one high risk group or for discordant couples trying to conceive and having heard of PrEP increased (all p-values <0.0001). Willingness to prescribe PrEP for MSM, PWID, persons who change partners frequently and for discordant couples not trying to conceive remained stable (p-values >0.5) but decreased for persons with an STD (p-value <0.004) and differed by primary care provider type (Figs 1 and 2).

Across the survey years 2009, 2010, and 2013, 58% of clinicians supported the use of state or federal government funds to provide PrEP for uninsured persons.

### Predictors of Willingness to Prescribe PrEP

In a multivariable association analysis, only five clinician factors were modestly predictive of willingness to prescribe PrEP to one or more risk populations (Table 3). Obstetrician/gynecologists were slightly more willing than family physicians, as were those clinicians in a group practice, compared to a solo practice. Clinicians in larger practices (>20 providers) were slightly less willing to prescribe PrEP than those in small practices (<5 providers), as were clinicians 45–54 years of age compared to those 55 years of age or older. Gender, race/ethnicity, and number of years in practice were not predictive. Having provided antiretrovirals for postexposure prophylaxis or for treatment of HIV infection was also predictive of willingness to prescribe PrEP. In a multivariable trend analysis, three physician characteristics, having provided antiretrovirals for postexposure prophylaxis or for treatment of HIV infection, age, and the interaction of age with years in practice were predictive of an increase in willingness to prescribe PrEP (adjusted p-value of <0.001).

**Table 3:** Association of Clinician Characteristics with Willingness to Prescribe PrEP: DocStyles 2009–2015, United States.

Characteristic	No. (%)	Univariate Prevalence Ratio (95% CI)	Multivariate Prevalence Ratio (95% CI)
Age (years)			
≥55	1708 (92.03)	Referent Group	Referent Group
45–54	2013 (90.43)	0.98 (0.96, 1.00)	0.98 (0.96, 1.00)*†
35–44	2476 (90.46)	0.98 (0.97, 1.00)	0.98 (0.95, 1.01)
20–34	647 (92.96)	1.01 (0.99, 1.04)	1.01 (0.97, 1.05)
Gender			
Male	4137 (90.51)	Referent Group	Referent Group
Female	2707 (91.95)	1.02 (1.00, 1.03)*	1.02 (1.00, 1.03)
Race/Ethnicity			
White	4849 (90.98)	Referent Group	Referent Group
Black	229 (92.71)	1.02 (0.98, 1.06)	1.01 (0.98, 1.05)
Hispanic	274 (89.25)	0.98 (0.94, 1.02)	0.98 (0.94, 1.02)
Asian	1153 (92.02)	1.01 (0.99, 1.03)	1.02 (1.00, 1.04)
Other	339 (89.68)	0.99 (0.95, 1.02)	0.99 (0.96, 1.03)
Clinician Specialty			
Family/General Practitioner	2473 (91.05)	Referent Group	Referent Group
Internist	2055 (89.70)	0.99 (0.97, 1.00)	0.98 (0.96, 1.00)
Obstetrician/Gynecologist	1169 (93.45)	1.03 (1.01, 1.05)*	1.04 (1.02, 1.06)*
Nurse Practitioner	1147 (91.25)	1.00 (0.98, 1.02)	1.00 (0.98, 1.02)
Clinician Work Setting			
Individual Outpatient Practice	1321 (90.05)	Referent Group	Referent Group
Group Outpatient Practice	4552 (91.44)	1.02 (1.00, 1.04)	1.02 (1.00, 1.04)
Hospital Inpatient Practice	971 (90.75)	1.01 (0.98, 1.03)	1.02 (0.99, 1.05)
Number of Clinicians in Practice			
≤5	3666 (91.28)	Referent Group	Referent Group
6–19	2054 (91.70)	1.00 (0.99, 1.02)	1.00 (0.98, 1.01)
≥20	1124 (89.28)	0.98 (0.96, 1.00)*†	0.97 (0.95, 0.99)*
Number Years in Practice			
>20	1937 (91.45)	Referent Group	Referent Group
11–20	2490 (90.84)	0.99 (0.98, 1.01)	1.00 (0.97, 1.02)
6–10	1723 (90.64)	0.99 (0.97, 1.01)	0.99 (0.97, 1.02)
0–5	694 (91.92)	1.01 (0.98, 1.03)	1.00 (0.97, 1.04)
Prescribed nPEP, oPEP, ARTs			
No	4467 (89.20)	Referent Group	Referent Group
Yes	2377 (94.81)	1.06 (1.05, 1.08)*	1.07 (1.06, 1.09)*

CI: Confidence Interval; No.: number of Yes respondents for the indicated characteristic.

* Confidence intervals indicate a statistically significant association at the 0.05 significance level.

† Some CIs that do not but might appear to include 1.0 as a result of rounding

Note: Univariate analyses test the association between the indicated characteristic and prescribing for at least one high risk group; multivariate analyses show the association between the indicated characteristic and prescribing for at least one high risk group while adjusting for all other characteristics listed in the table; Pediatricians were not solicited to respond to this question.

### PrEP Implementation Factors

Eight to ten percent had read one or more of the interim guidance documents issued by CDC published in 2011, 2012, and 2013. However, in 2014, 17% had read the final clinical practice guidelines increasing to 22% in 2015. Clinicians most commonly reported that formal CDC or PHS guidelines (52%) would have the greatest influence about prescribing PrEP, followed by a US Preventive Services Task Force recommendation (19%) or a recommendation from their specialty professional association (15%). Few would be guided primarily by American Medical Association or National Medical Association clinical practice statements (6%), state or local public health department guidance (5%), or a practice statement from their clinical organization (3%).

In response to several PrEP knowledge true/false questions presented on the surveys in 2012, 2013, and 2015, clinicians reported limited knowledge. For four of the questions the most common answer was “don’t know” (64–68%). Incorrect response rates were greater than correct responses for 2 questions: Atripla is recommended, safe and effective for PrEP (22% true v 11% false) and follow-up HIV testing should be done at least once a year for those on PrEP (58% true vs 5% false). Correct response rates were greater than incorrect responses for 4 questions: heterosexuals do not need hepatitis B screening before starting PrEP (8% true v 57% false, asked in 2013 and 2015), a negative HIV test result should be documented before prescribing PrEP (55% true vs 10% false), PrEP with Truvada is indicated during pregnancy for an uninfected woman with an HIV-infected sexual partner (25% true vs 7% false), and PrEP is safe and effective when taken just before and after sex instead of daily (26% false vs 11% true). In 2015, of 1501 respondents, 3 clinicians answered all questions incorrectly and 5 clinicians answered all questions correctly.

Most clinicians (83%) expressed interest in participating in online CME training on one of more of the following topics: screening and selecting patients for PrEP (73%), managing side effects and monitoring for adverse events (59%), brief risk-reduction counseling (57%), brief medication adherence counseling (53%), taking a brief sexual history (49%), and billing for PrEP-related services (48%).

When asked how they would provide recommended risk-reduction counseling to PrEP patients if it were reimbursable, 37% would do the counseling themselves, 19% would have a health education or other staff member provide the counseling, 17% would send the patient to a dedicated counseling site in their clinic system, and 17% would refer for counseling in the community. A majority of clinicians (59%) felt that counseling should be reimbursed at $50-$100 while 22% felt that $101–200 was appropriate.

### Pharmacy Services

In the 2012 survey only, pharmacists were asked about pharmacy services that could be used for PrEP medication dispensing and other potential PrEP-related support. Among these pharmacists, 66% reported that they currently provide reimbursable medication therapy management (MTM) services, 43% that they currently provide some services under a collaborative practice agreement, and 57% that they would be interested in providing on-site HIV testing for pharmacy clients.

This is the first multi-year survey to assess knowledge and attitudes about PrEP among large national samples of primary care clinicians in the US. In the DocStyles survey waves conducted in 2009 and 2010, prior to publication of the results of the first trial documenting its effectiveness and safety [2], awareness of PrEP was low among primary care clinicians. In the years after trial results were published, awareness rose substantially.

The patient population for whom PrEP is indicated, i.e., persons without HIV infection, are not usually receiving health care from infectious disease or HIV treatment specialists. They often may not wish to seek health care in HIV treatment clinics because of a concern that, if seen there by persons who know them, they will be thought to have HIV-infection. Primary care clinicians providing general health care for persons without HIV infection may therefore play a significant role in PrEP delivery [17].

A qualitative study of HIV care providers proposed a “purview paradox” in which HIV specialists thought primary care providers were best positioned to provide PrEP, but HIV care providers were most capable to do it [18]. However, a recent study that included convenience samples of both HIV and non-HIV providers attending HIV conferences in California and New York, each type of provider (HIV, non-HIV) felt that they were best suited to provide PrEP. This study also reported that HIV providers had more knowledge about PrEP and that knowledge was a strong predictor of future intent to prescribe PrEP [19]. An earlier study of STD and family planning clinic providers also found higher willingness to provide PrEP among clinicians with higher knowledge scores [20]. In the DocStyles survey, clinicians reported low exposure to any of the CDC interim guidance issued in 2011–2013 [21–23] (8–10%) and limited knowledge of PrEP on true false questions with high rates of “don’t know” as a response and very few answering all 5 questions correctly. Clinicians were aware of their knowledge deficits and indicated substantial interest in CME for all topics presented. They also indicated they would be influenced by formal CDC/PHS guidelines. These were issued in May 2014 [24].

In all DocStyles survey years, both primary care clinicians initially aware of PrEP and those initially unaware but provided with information about it, reported high levels of willingness to prescribe PrEP to at least one subgroup of persons at substantial risk for HIV acquisition. In addition, support for public funding of PrEP for those who could not otherwise afford it was consistently high among clinicians across survey years.

There are limitations to this study. While the effectiveness described on the surveys ranged from 75% in 2009 and 2010 to ~90% among adherent users in 2012–2015, the high and stable estimated willingness to prescribe PrEP reported across the five waves of the survey when this was assessed may or may not be a realistic approximation of primary care clinicians’ attitudes in the US in this early phase of introducing PrEP as a clinical HIV prevention method.

While DocStyles is a large, national survey that includes a diverse group of primary care clinicians, it is not a fully representative survey sample. However, the selection of invited participants using quota sampling has been found to include participants that were demographically comparable (gender, age, average years in practice) with physicians in the AMA Masterfile (unpublished data, Porter Novelli, DocStyles 2009 Methods, Washington DC, 2009).

Additional limitations are the change in sampling frame that occurred between the 2010 and 2012 surveys and the small amount of information available about the patient populations served by the respondents. We did not assess the proportion of the respondent’s patients who have risk factors that are potential indications for PrEP, e.g., MSM, PWID, persons in HIV-discordant sexual partnerships, or sexually active persons with frequent bacterial STIs diagnosed.

It is critical that primary care providers with such patients are knowledgeable about and willing to prescribe PrEP. A recent qualitative study found that clinical and non-clinical providers in substance abuse treatment centers in New York City had limited awareness of PrEP’s effectiveness for PWID [3] (11% of respondents) but when it was explained to them, identified challenges to incorporating it into their services, as well as receptivity to learning about its delivery to clients they identified as likely to benefit from it [25]. A recent survey of women’s health clinicians attending a regional HIV conference found that 66–72% (depending on the sex of the uninfected partners) had discussed PrEP with an HIV-discordant couple interested in pregnancy conception [26]. For primary care clinicians to successfully incorporate PrEP into their practices, it will be important to learn applicable lessons from adoption of other preventive innovations in clinical settings [27]. This should include attention to factors affecting both their commitment (e.g., awareness, anticipated perceptions, role congruence) and their perceived capacity (e.g., service delivery congruence, role support, skills efficacy) to introduce a new method.

## Conclusions

As PrEP is introduced into clinical practice and its availability is scaled-up, it will be important to continue to monitor clinician knowledge, attitudes, and practices for PrEP and other clinically-delivered HIV prevention interventions. This will facilitate creating materials and methods for both providers and their patients that will increase awareness of PrEP and its prescription when indicated with effective support for the medication adherence necessary to achieve significant reductions in the risk of HIV acquisition.

## References

[1] Centers for Disease Control and Prevention. Estimated HIV incidence in the United States, 2007–2010. HIV Surveillance Supplementatl Report [Internet]. 2012 20 4 2013; 17(4). Available from: http://www.cdc.gov/hiv/surveillance/resources/reports/2010supp_vol17no4/index.htm.

[2] Grant RM, Lama JR, Anderson PL, McMahan V, Liu AY, Vargas L, et al Preexposure chemoprophylaxis for HIV prevention in men who have sex with men. The New England journal of medicine. 2010;363(27):2587–99. 10.1056/NEJMoa1011205 21091279 PMC3079639

[3] Choopanya K, Martin M, Suntharasamai P, Sangkum U, Mock PA, Leethochawalit M, et al Antiretroviral prophylaxis for HIV infection in injecting drug users in Bangkok, Thailand (the Bangkok Tenofovir Study): a randomised, double-blind, placebo-controlled phase 3 trial. Lancet. 2013;381(9883):2083–90. 10.1016/S0140-6736(13)61127-7 23769234

[4] Baeten JM, Donnell D, Ndase P, Mugo NR, Campbell JD, Wangisi J, et al Antiretroviral Prophylaxis for HIV Prevention in Heterosexual Men and Women. New England Journal of Medicine. 2012;367(5):399–410. 10.1056/NEJMoa1108524 22784037 PMC3770474

[5] Thigpen MC, Kebaabetswe PM, Paxton LA, Smith DK, Rose CE, Segolodi TM, et al Antiretroviral Preexposure Prophylaxis for Heterosexual HIV Transmission in Botswana. New England Journal of Medicine. 2012;367(5):423–34. 10.1056/NEJMoa1110711 22784038

[6] Young I, McDaid L. How acceptable are antiretrovirals for the prevention of sexually transmitted HIV?: a review of research on the acceptability of oral pre-exposure prophylaxis and treatment as prevention. AIDS and behavior. 2014;18(2):195–216. 10.1007/s10461-013-0560-7 23897125 PMC3905168

[7] Mimiaga MJ, Case P, Johnson CV, Safren SA, Mayer KH. Preexposure antiretroviral prophylaxis attitudes in high-risk Boston area men who report having sex with men: limited knowledge and experience but potential for increased utilization after education. Journal of acquired immune deficiency syndromes (1999). 2009;50(1):77–83. 19295337 10.1097/QAI.0b013e31818d5a27 PMC2659469

[8] Voetsch AC, Heffelfinger JD, Begley EB, Jafa-Bhushan K, Sullivan PS. Knowledge and use of preexposure and postexposure prophylaxis among attendees of minority gay pride events, 2005 through 2006. JAIDS Journal of Acquired Immune Deficiency Syndromes. 2007;46(3):378–80. 18090305 10.1097/QAI.0b013e3181576874

[9] Golub SA, Kowalczyk W, Weinberger CL, Parsons JT. Preexposure prophylaxis and predicted condom use among high-risk men who have sex with men. Journal of acquired immune deficiency syndromes (1999). 2010;54(5):548. 20512046 10.1097/QAI.0b013e3181e19a54 PMC2908204

[10] Khawcharoenporn T, Kendrick S, Smith K. HIV risk perception and preexposure prophylaxis interest among a heterosexual population visiting a sexually transmitted infection clinic. AIDS patient care and STDs. 2012;26(4):222–33. 10.1089/apc.2011.0202 22404427

[11] Whiteside YO, Harris T, Scanlon C, Clarkson S, Duffus W. Self-perceived risk of HIV infection and attitudes about preexposure prophylaxis among sexually transmitted disease clinic attendees in South Carolina. AIDS patient care and STDs. 2011;25(6):365–70. 10.1089/apc.2010.0224 21470046

[12] Arnold EA, Hazelton P, Lane T, Christopoulos KA, Galindo GR, Steward WT, et al A qualitative study of provider thoughts on implementing pre-exposure prophylaxis (PrEP) in clinical settings to prevent HIV infection. PloS one. 2012;7(7):e40603 10.1371/journal.pone.0040603 22792384 PMC3394704

[13] White JM, Mimiaga MJ, Krakower DS, Mayer KH. Evolution of Massachusetts physician attitudes, knowledge, and experience regarding the use of antiretrovirals for HIV prevention. AIDS patient care and STDs. 2012;26(7):395–405. 10.1089/apc.2012.0030 22694239 PMC3432573

[14] Tellalian D, Maznavi K, Bredeek UF, Hardy WD. Pre-exposure prophylaxis (PrEP) for HIV infection: results of a survey of HIV healthcare providers evaluating their knowledge, attitudes, and prescribing practices. AIDS patient care and STDs. 2013;27(10):553–9. 10.1089/apc.2013.0173 . 24053478

[15] Puro V, Palummieri A, De Carli G, Piselli P, Ippolito G. Attitude towards antiretroviral Pre-Exposure Prophylaxis (PrEP) prescription among HIV specialists. BMC infectious diseases. 2013;13:217 10.1186/1471-2334-13-217 23672424 PMC3658955

[16] Mullins TLK, Lally M, Zimet G, Kahn JA. Clinician Attitudes Toward CDC Interim Pre-Exposure Prophylaxis (PrEP) Guidance and Operationalizing PrEP for Adolescents. AIDS patient care and STDs. 2015;29(4):193–203. 10.1089/apc.2014.0273 25692683 PMC4378662

[17] Thrun MW. Opportunity knocks: HIV prevention in primary care. LGBT Health. 2014;1(2):75–8. 10.1089/lgbt.2014.0007 26789615

[18] Krakower D, Ware N, Mitty JA, Maloney K, Mayer KH. HIV providers’ perceived barriers and facilitators to implementing pre-exposure prophylaxis in care settings: A qualitative study. AIDS and behavior. 2014;18(9):1712–21. 10.1007/s10461-014-0839-3 24965676 PMC4127184

[19] Blumenthal J, Jain S, Krakower D, Sun X, Young J, Mayer K, et al Knowledge is Power! Increased Provider Knowledge Scores Regarding Pre-exposure Prophylaxis (PrEP) are Associated with Higher Rates of PrEP Prescription and Future Intent to Prescribe PrEP. AIDS and behavior. 2015:1–9. 25616837 10.1007/s10461-015-0996-z PMC4417031

[20] Tripathi A, Ogbuanu C, Monger M, Gibson JJ, Duffus WA. Preexposure prophylaxis for HIV infection: healthcare providers' knowledge, perception, and willingness to adopt future implementation in the southern US. Southern medical journal. 2012;105(4):199–206. 10.1097/SMJ.0b013e31824f1a1b 22475669

[21] Smith DK, Grant RM, Weidle PJ, Lansky A, Mermin J, Fenton KA. Interim Guidance: Preexposure Prophylaxis for the Prevention of HIV Infection in Men Who Have Sex With Men (Reprinted from MMWR, vol 60, pg 65–68, 2011). Jama-Journal of the American Medical Association. 2011;305(11):1089–91. .

[22] Centers for Disease Control and Prevention. Interim guidance for clinicians considering the use of preexposure prophylaxis for the prevention of HIV infection in heterosexually active adults. Morbidity and Mortality Weekly Reports. 2012;61(31):586–90. 22874836

[23] Centers for Disease Control and Prevention. Update to Interim Guidance for Preexposure Prophylaxis (PrEP) for the Prevention of HIV Infection: PrEP for Injecting Drug Users. MMWR. 2013;62(23):463–5. 23760186 PMC4604844

[24] Centers for Disease Control and Prevention; US Public Health Service. Preexposure prophylaxis for the prevention of HIV infection in the United States—2014: a clinical practice guideline2014 28 May 2014:[1–67 pp.]. Available from: http://www.cdc.gov/hiv/pdf/guidelines/PrEPguidelines2014.pdf.

[25] Spector AY, Remien RH, Tross S. PrEP in substance abuse treatment: a qualitative study of treatment provider perspectives. Substance abuse treatment, prevention, and policy. 2015;10(1):1. 10.1186/1747-597X-10-1 PMC4298071 25575428

[26] Rahangdale L, Richardson A, Carda-Auten J, Adams R, Grodensky C. Provider Attitudes toward Discussing Fertility Intentions with HIV-Infected Women and Serodiscordant Couples in the USA. Journal of AIDS & clinical research. 2014;5(6):1000307. 25221730 10.4172/2155-6113.1000307 PMC4160891

[27] Laws RA, Kemp LA, Harris MF, Davies GP, Williams AM, Eames-Brown R. An exploration of how clinician attitudes and beliefs influence the implementation of lifestyle risk factor management in primary healthcare: a grounded theory study. Implementation Science. 2009;4(1):66. 19825189 10.1186/1748-5908-4-66 PMC2770564

